# A rare case report of delayed thyroxine hypersensitivity: Challenges and management strategies

**DOI:** 10.51866/cr.734

**Published:** 2025-05-10

**Authors:** A Kari Halimah Hana, Ahmad Saharuddin

**Affiliations:** 1 MD, MMed (FamMed), Department of Family Medicine, Hospital Canselor Tuanku Muhriz UKM, Jalan Yaacob Latif, Bandar Tun Razak, Kuala Lumpur, Malaysia. E-mail: saha@ppukm.ukm.edu.my; 2 MBChB, Department of Family Medicine, Hospital Canselor Tuanku Muhriz UKM, Jalan Yaacob Latif, Bandar Tun Razak, Kuala Lumpur, Malaysia.

**Keywords:** Hypersensitivity, Thyroxine, Hypothyroidism

## Abstract

Herein, we present the case of a 45-year-old woman with a history of Graves’ disease who underwent radioactive iodine therapy and was prescribed different oral preparations of thyroxine. Despite initial tolerance to the medication, she developed hypersensitivity reactions 3 months after consumption, which presented as urticarial rashes, erythematous maculopapular eruptions and angio-oedema. She tried a different preparation of thyroxine accompanied by antihistamines but continued having persistent reactions. This led to her discontinuation of thyroxine, resulting in overt hypothyroidism with secondary dyslipidaemia and weight gain. A diagnosis of delayed thyroxine hypersensitivity was made based on history, clinical assessment and supportive evidence from the Naranjo Adverse Drug Reaction Probability Scale.

## Introduction

Hypothyroidism is a common disorder often managed effectively with oral thyroxine by primary care doctors. However, treatment can be compromised by thyroxine hypersensitivity, leading to complications of hypothyroidism. Drug hypersensitivity is an immune-mediated response to a drug in a sensitised patient.^1^ This case report describes a rare occurrence of a delayed thyroxine hypersensitivity reaction and its subsequent management.

## Case presentation

This case report is of a 45-year-old woman with a history of Graves’ disease who underwent radioactive iodine (RAI) therapy in 2019 due to frequent relapses while on antithyroid medications. Post-RAI therapy, she received treatment for hypothyroidism with oral levothyroxine 100 pg once daily. After 3 months, she developed an urticarial rash on her upper limbs, torso, back and inguinal regions. Her medication was changed to a different preparation of levothyroxine 50 pg once daily. However, the rash persisted, and she had additional erythematous maculopapular eruptions on her face and swelling of her lips and conjunctivae. She visited a primary care clinic and received intravenous steroids before being admitted for observation under the dermatology and endocrinology teams.

Following the resolution of the allergic reactions, she was restarted on levothyroxine 50 pg daily with loratadine 20 mg twice a day. She initially tolerated the medications, but 3 months later, the rashes had recurred following the same pattern as the initial presentation. She was advised to continue the same treatment, as the reaction was considered mild. Nevertheless, she unilaterally chose to cease taking the medications. She returned to the primary care clinic in 2023 with hypothyroid symptoms, including numbness, cold intolerance and a 5-kg weight gain. She had stopped taking levothyroxine for 6 months due to fear of allergic reactions.

Laboratory investigations revealed overt hypothyroidism (free T4 level: 8.39 pmol/L [normal range: 9-19.05 pmol/L]; thyroid-stimulating hormone level: 10.31 ulU/mL [normal range: 0.35-4.94 uIU/mL]) and worsening lipid profile (total cholesterol level: from 5.08 to 6.32 mmol/L; low-density lipoprotein level: from 3.51 to 4.47 mmol/L) but with normal fasting blood sugar and vitamin B12 levels.

The components of her previous levothyroxine preparations were compared, and no common ingredient, other than thyroxine, was found to cause her hypersensitivity. A diagnosis of delayed thyroxine hypersensitivity was made based on the temporal relationship between thyroxine use and the onset and resolution of her symptoms. The diagnosis was supported by a score of 9 on the Naranjo Adverse Drug Reaction Probability Scale.

She was referred back to the endocrinology team for thyroxine induction of drug tolerance (IDT). Thyroxine was reintroduced with concurrent use of antihistamines, and she has been able to tolerate it for at least 5 months.

## Discussion

This case report highlights the complexity of managing hypothyroidism complicated by delayed thyroxine hypersensitivity. Thyroxine hypersensitivity is uncommon, with only few case reports ever documented. Delayed thyroxine hypersensitivity is even rarer, making this the second reported case globally and the first in the Asian region.^2-5^ The present case resembles a prior report of urticarial rashes appearing a few months after initiating various thyroxine preparations and resolving upon discontinuation. The case was successfully treated with IDT.^3^ However, our patient presented an additional feature of angio-oedema during the change to a different thyroxine preparation.

In general, an adverse drug reaction (ADR) is any unwanted reaction secondary to a drug and can be classified into a predictable or an unpredictable reaction. Predictable ADRs of thyroxine include tachycardia, palpitations, anxiety, diarrhoea and symptoms of hyperthyroidism. These reactions are dose-dependent and arise from the therapeutic action of thyroxine or its known side effects. Meanwhile, unpredictable ADRs are immune-mediated and not related to their pharmacological effects, also known as drug hypersensitivity reactions (DHRs). Symptoms may include skin rashes, angio-oedema and anaphylaxis.^6^ DHRs to thyroxine can be attributed to either the active or inactive components such as preservatives and fillers. A multicentre review conducted in the United States of America found that the prevalence of ADRs to thyroxine was 0.3%, with 42.5% being DHRs.^7^ Our patient was likely to have hypersensitivity towards the active ingredient, as she developed the same reaction to both thyroxine preparations and no other common ingredients were identified.

There is no clear guideline on the management of thyroxine hypersensitivity, but options include skin testing, drug provocation test (DPT) and IDT.^7^ Skin testing is preferred for immediate type 1 hypersensitivity, while DPT is suitable for patients with a low likelihood of drug allergy.^6^ Our patient had a delayed instead of an immediate hypersensitivity, with a high probability of drug allergy based on a score of 9 on the Naranjo Adverse Drug Reaction Probability Scale. This scale is a validated tool used to determine the likelihood of an ADR. It is based on 10 questions, scored as follows: a score of ≥9 indicates a definite likelihood of ADRs; 5-8, probable likelihood; 1-4, possible likelihood; and ≤0, doubtful likelihood.^4,6,8^ The additional angio-oedema reaction also made DPT a less favourable option for our patient. Thus, an IDT was thought to be necessary to create tolerance and safely continue thyroxine, as reported in a prior case.^3^ The concomitant use of thyroxine with antihistamines has also been reported as an option for treatment.^4^

IDT, previously called drug desensitisation, is a procedure that modifies the immune response to allow a sensitised person to safely continue a medication.^9^ Reports of thyroxine IDT have been documented in both inpatient and outpatient settings.^3,5^ Inpatient IDT was performed in immediate hypersensitivity with 0.01 pg of oral thyroxine, doubled every 30 minutes to reach a total of 110 pg over 7 hours.^5^ Outpatient IDT was performed in delayed hypersensitivity with 0.075 pg thyroxine daily, with weekly increments to reach 75 pg in 7 weeks.^3^ This case was referred for inpatient IDT for close monitoring given her history of angio-oedema.

## Conclusion

Primary care doctors play a crucial role in diagnosing and managing thyroxine hypersensitivity. The discrepancy between our referral and the endocrinology team’s management highlights the variability in approaching this rare condition, also reflected in the varied management in prior case reports. This emphasises the complexity of this condition and suggests a standardised guideline.
